# Induction of Overt Menstruation in Intact Mice

**DOI:** 10.1371/journal.pone.0032922

**Published:** 2012-03-07

**Authors:** Marion Rudolph, Wolf-Dietrich Döcke, Andrea Müller, Astrid Menning, Lars Röse, Thomas Matthias Zollner, Isabella Gashaw

**Affiliations:** 1 Target Discovery, Global Drug Discovery, Bayer HealthCare, Berlin, Germany; 2 Target Research Group Women's Healthcare, Global Drug Discovery, Bayer HealthCare, Berlin, Germany; Clermont Université, France

## Abstract

The complex tissue remodeling process of menstruation is experienced by humans and some primates, whereas most placental mammals, including mice, go through an estrous cycle. How menstruation and the underlying mechanisms evolved is still unknown. Here we demonstrate that the process of menstruation is not just species-specific but also depends on factors which can be induced experimentally. In intact female mice endogenous progesterone levels were raised by the induction of pseudopregnancy. Following an intrauterine oil injection, the decidualization of the endometrium was reliably induced as a prerequisite for menstruation. The natural drop of endogenous progesterone led to spontaneous breakdown of endometrial tissue within an average of 3 days post induction of decidualization. Interestingly, morphological changes such as breakdown and repair of the endometrial layer occurred in parallel in the same uterine horn. Most importantly, endometrial breakdown was accompanied by vaginally visible (overt) bleeding and flushing out of shed tissue comparable to human menstruation. Real-time PCR data clearly showed temporal changes in the expression of multiple factors participating in inflammation, angiogenesis, tissue modulation, proliferation, and apoptosis, as has been described for human menstruating endometrium. In conclusion, human menstruation can be mimicked in terms of extravaginally visible bleeding, tissue remodeling, and gene regulation in naturally non-menstruating species such as intact female mice without the need for an exogenous hormone supply.

## Introduction

Although menstruation is at least as old as the human species, knowledge of the underlying mechanisms is limited. Its physiology has rarely been studied, and this is partly due to a lack of appropriate animal models. Apart from humans very few species of Old World monkeys and apes experience a menstrual cycle. This includes the flushing out of endometrial tissue and blood from the uterus to the vagina, clearly visible as overt menstruation [1], [2]. Two menstruating species of non-primates have been described - the elephant shrew and the bat [3], [4]. The estrous cycle is much more common in placental mammals (such as dogs or rodents) than the menstrual cycle. The former is characterized by complete reabsorption of the endometrial lining, which is not externally visible.

Both reproduction cycles involve proliferation of stromal cells and ovulation, followed by the formation of the progesterone-producing corpus luteum in the ovary. However, in the menstrual cycle endometrial stromal cells differentiate into decidual cells in response to the rapidly increasing progesterone level despite the absence of a blastocyst. In contrast, in the estrous cycle decidualization occurs only after conception, e.g. in mice it starts on day 4 post coitum, when endometrial stromal cells surround the implanting blastocysts. The decidua provides a vascular network for nutrition and gas exchange for the developing embryo if implantation occurs before a functional placenta is established [5], [6]. In both the menstrual and the estrous cycle the absence of implantation induces the degeneration of the corpus luteum in the ovary and subsequently the progesterone level drops. This triggers the breakdown of endometrial tissue and the flushing of shed endometrium and blood in human overt menstruation, or the reabsorption of the endometrial lining in the estrous cycle.

Consequently, common laboratory animals such as mice or rats cannot be used to directly study the mechanisms of overt menstruation as it occurs in humans. In this regard, Finn and Pope described in 1984 a mouse model that mimics menstruation-like processes: ovariectomized mice treated with a special hormone schedule (exogenous progesterone and estrogen) showed decidualization after intrauterine oil injection, endometrial breakdown after progesterone withdrawal, and repair thereafter [7]. However, it has to be considered that utilizing ovariectomized mice combined with an artificial hormone supply excludes any natural impact of ovarian hormones, estrogen and progesterone, which are essential to govern the endometrial functions in human menstruation. Surprisingly, even though the intrinsic drawbacks of this model have been well recognized, very little work has been done to develop it further. In recent studies, the artificial exogenous hormone supply was still necessary and visible bleeding and shed tissue comparable to that seen in women has never been observed in mice [7], [8], [9], [10], [11], [12]. There is therefore still a great need for a model that mimics human menstruation. Against this background it first has to be clarified why certain species repel rather than reabsorb endometrial tissue and which mechanisms turn a non-menstruating species into an overtly menstruating species. Among other theories, menstruation has been proposed as a mechanism to protect the uterus from sperm-borne pathogens [2]. Another hypothesis suggests that repelling or reabsorbing the endometrial lining might be less costly than keeping it in an active metabolic state by luteal maintenance [13]. Whether expulsion or absorption takes place might be determined by the amounts of tissue and blood: for large quantities of blood and tissue shedding and flushing might be more economical [13].

To prove whether overt bleeding is determined by the quantity of differentiated endometrial tissue, or whether it is restricted to a few species the feasibility of inducing overt bleeding in mice was tested. Intact pseudopregnant mice were used to exploit intrinsic hormonal changes largely comparable to the human situation. The transferability to human menstruation was verified by investigating bleeding intensity and endometrial gene expression and by histological examination of the uteri. Importantly, in mice with decidualized endometrium the spontaneous drop of endogenous progesterone levels was sufficient to induce menstruation-like processes including the extravaginally visible bleeding typically seen in overt menstruation.

## Materials and Methods

### Mice

Female BALB/c mice aged 8 to 10 weeks were purchased from Taconic or Charles River Laboratories. Vasectomized Swiss Webster males older than 10 weeks were provided by Taconic. Mice were kept in a light/dark cycle of 12 h/12 h under pathogen-free conditions in the animal facility of Bayer Pharma AG, Berlin. All experiments were performed in strict compliance with company, regional, and federal guidelines for the use of laboratory animals. The study was approved by the Regional Office for Health and Social Affairs in Berlin (protocol A0229/10) and the company review board, and all efforts were made to minimize suffering.

### Pseudopregnancy, decidualization, and tissue collection

Two days prior to mating with vasectomized Swiss Webster males, female BALB/c mice were placed on the litter of males for 48 h to induce proestrus (the Whitten effect) [14]. After mating overnight, pseudopregnant female mice were selected based on the presence of a vaginal plug. Decidualization was induced by intrauterine injection of 100 µl sesame oil (Sigma-Aldrich, Germany) on day 4 of pseudopregnancy. Uteri were collected from mice in proestrus, on day 4 of pseudopregnancy and after decidualization on days 5, 6, 7, 8, 9, 10 and 12 of pseudopregnancy, and wet weights were determined. The uteri were documented macroscopically by photographing with a Canon PowerShot A640 (Canon, Germany) using a 2000 C stereo microscope and AxioVision 4.6.3.0 software (both Zeiss, Germany).

### Mifepristone administration

Mice with decidualized endometrium were treated subcutaneously on the evening of day 6 of pseudopregnancy with 10 mg/kg mifepristone (Bayer Pharma AG, Germany) in 5% v/v ethanol (Merck, Germany) and 95% peanut oil (Sigma-Aldrich, Germany).

### Histology

Samples of uterine tissues were fixed in PBS-buffered 4% formaldehyde. Paraffin infiltration of uterine tissue was done with an ASP200 vacuum tissue processor (Leica, Germany) followed by embedding with a modular paraffin embedding system (Medax, Germany). Paraffin sections (rotary microtomes; Leica, Germany; 4 µm thick) were stained with hematoxylin (Merck, Germany) and eosin (Sigma-Aldrich, Germany; H&E staining). Slides were scanned in and analyzed with the Mirax midi slide scanner and Mirax viewer software (both Zeiss, Germany) using a 2.5× magnification.

### ELISA

Samples of blood were collected from the retrobulbar venous plexus at the times indicated during the experiments or from the inferior vena cava at necropsy. Serum progesterone levels were determined using a progesterone-specific ELISA (GenWay, USA).

### Monitoring of bleeding

**Figure 1:**
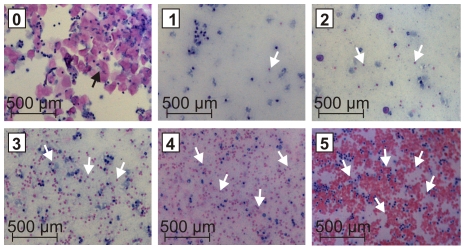
Scoring system for the determination of bleeding intensity on the basis of hematoxylin and eosin (H&E) staining of vaginal lavage fluid. The increase in erythrocytes correlates with the increase in the indicated score (from 0 = no erythrocytes, no bleeding to 5 = large amounts of erythrocytes, heavy bleeding). Black arrows indicate eosin-stained squamous epithelial cells and white arrows eosin-stained erythrocytes.

**Figure 2:**
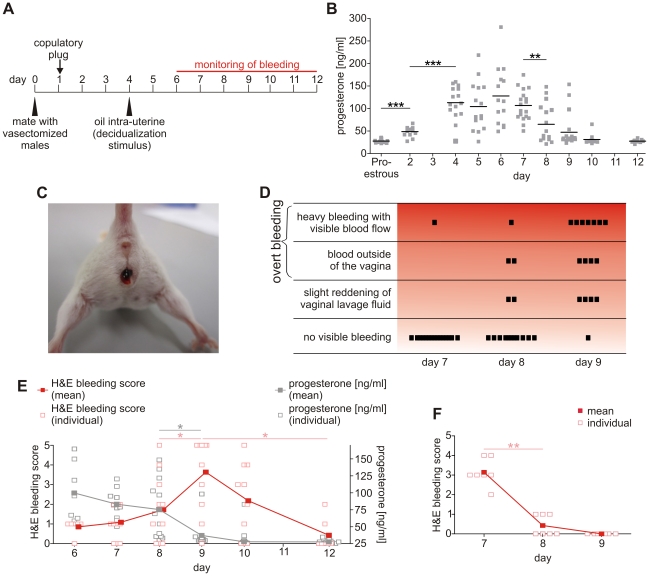
A drop in endogenous progesterone levels induces overt, extravaginally visible bleeding. **A:** Experimental setup: Female mice were mated with vasectomized male mice (day 0). Pseudopregnant female mice were identified by vaginal plugs the next day. On day 4, endometrial decidualization was triggered by intra-uterine injection of sesame oil. The bleeding was monitored visually (detection of extravaginal blood) and microscopically (in H&E stained vaginal lavage fluid) from day 6 until necropsy. **B:** Systemic progesterone concentrations (mean) were determined by ELISA in sera from mice treated according to Figure 2A. The Figure shows pooled data from two independent experiments (N≥10). **C:** Representative photograph of a mouse bleeding visibly extravaginally 4 days after decidualization on day 8 of pseudopregnancy. **D:** Visual determination of bleeding intensity of pseudopregnant mice with decidualized endometrium over 3 days from day 7 to day 9 (N = 16). **E:** Bleeding intensity (H&E bleeding scores; left axis) and pattern of serum progesterone levels (right axis) over time (open square = result for one individual mouse; filled squares = mean score for all mice in the group; N≥7; red = H&E bleeding score; gray = progesterone concentration). Data were collected in a single experiment. **F:** Bleeding scores of mifepristone-treated, pseudopregnant mice from day 7 to day 9 (treatment started on the evening of day 6; 10 mg/kg sc; open/filled squares see Figure 2E; N = 7). The unpaired two-tailed Student's test was used for statistical analysis of the progesterone data (B, E), the two-tailed Mann-Whitney test for the ordinal H&E scores (*p≤0.05, **p≤0.01, ***p≤0.001).

Bleeding intensity was determined by vaginal lavage carried out with 40 µl PBS every day from day 6 until day 12. Three microliters of lavage fluid was plated on a slide, dried and H&E staining was performed. Bleeding intensity was classified on the basis of the scoring system depicted in Figure 1. In addition, the bleeding was visually determined and staged into (1) no visible bleeding, (2) slight reddening of vaginal lavage fluid, indicative of overt menstruation, (3) blood outside of the vagina, or (4) heavy bleeding with visible blood flow (Figure 2D).

### Quantitative RT-PCR

Endometrium was collected from mice in proestrus and on days 4, 9, and 14 after induction of pseudopregnancy by scraping the endometrial layer into Lysing Matrix D tubes (MP Biomedicals, Germany) containing RLT buffer (Qiagen, Germany) and stored at −80°C. For mRNA extraction the tissue was lysed with FastPrep-24 (MP Biomedicals, Germany) and the RNeasy Mini Kit (Qiagen, Germany) was used following the manufacturer's guidelines. Purified RNA was qualified and quantified with the Agilent bioanalyzer 2100 system with a RNA 6000 nano assay kit (Agilent Technologies, Germany). cDNA was synthesized using the high capacity RNA-to-cDNA master mix and GeneAmp® 9700 PCR System (Applied Biosystems, Germany). RT-PCR with cDNA was performed on a 7900HT sequence detection system (Applied Biosystems, Germany) under the thermal conditions: 10 min at 95°C, 40 cycles, 15 sec at 95°C and 1 min at 60°C.

Hprt1, Hmox1, Il15 and Tgfb3 were analyzed using assays on demand and Cxcl12, Tnf, Ifng, Il1b, Il6, Il18, Vegfa, Tgfb1, Ptgs1, Fn1, Igf1, Mki67, Bax, Bcl2, Pecam1, Vim, Krt19, Krt8, Ptgs1 and Ptgs2 were analyzed using low-density array microfluidic cards (both Applied Biosystems, Germany). The reactions were performed using a universal PCR master mix without UNG (Eurogentec, Germany). Expression of target genes was quantified in relative terms as fold expression of the housekeeping gene, HPRT.

### Statistics

Levels of progesterone and relative mRNA were considered to be normally distributed. All samples were independent. For statistical analysis of mean differences between measurements between days, unpaired two-tailed Student's t-tests (GraphPad Prism, GraphPad Software, Inc., La Jolla, USA) were carried out with a significance level of 0.05. H&E scores are ordinal and thus do not follow a Gaussian distribution. Two-tailed Mann-Whitney tests were used to analyze differences between scores on two days. Spearman's rank correlation coefficient was calculated and tested for significant deviation from 0 with α = 0.05 to determine the correlation between uterine weight and H&E scores. Test results are given as p-values; significant values are shown as *p≤0.05 and **p≤0.01, highly significant values as ***p≤0.001. Data are shown as means.

## Results

### Pseudopregnant mice with decidualized endometrium display overt bleeding induced by the endogenous drop of progesterone

The focus of our study was to evaluate whether non-menstruating species like mice can be adapted to display overt bleeding. For this purpose, we developed the experimental scheme displayed in Figure 2A which is based on mating female mice with vasectomized males. The high levels of progesterone which are necessary for decidualization were achieved by inducing pseudopregnancy. Subsequently, decidualization was triggered by intrauterine injection of sesame oil on day 4. Large quantities of sesame oil (100 µl) achieved uniform uterine distribution, as has been demonstrated in experiments using colored oil (data not shown). This induced decidualization in both uterine horns.

A significant increase in endogenous progesterone levels was observed in mice on day 2 of pseudopregnancy compared to mice in proestrus (Figure 2B). High levels of progesterone were detected from day 4 to day 7 of pseudopregnancy and thus at the time of decidualization. A significant drop in serum progesterone concentrations was evident between day 7 and day 8. During this period we noticed the onset of very heavy bleeding visible outside the vagina (Figure 2C) in more than 80% of the pseudopregnant mice with decidualized endometrium observed over a period of 3 days (day 7 to day 9 of pseudopregnancy; Figure 2D). Detailed analysis of bleeding intensity based on the H&E bleeding score in vaginal lavage fluid indicated that the drop in progesterone level coincided with the onset of bleeding (Figure 2E). In addition, administration of the progesterone receptor antagonist mifepristone on day 6 caused a rapid onset of bleeding: 6 of the 7 mice that were given mifepristone displayed heavy bleeding with an H&E score greater than or equal to 3 on day 7 and no bleeding on day 9. In comparison, none of the mice not given mifepristone had an H&E bleeding score greater than 2 on day 7, whereas on day 9 more than two-thirds of the mice (8 out of 11) were scored at 3 or higher (Figure 2E). Administration of mifepristone thus shifted the peak of bleeding from day 9 to day 7. Hence, clearly visible bleeding is inducible in intact pseudopregnant female mice with decidualized endometrium. The onset of bleeding was caused by the drop in endogenous progesterone levels. This was additionally confirmed by experimental withdrawal of progesterone signaling by administering mifepristone.

### Tissue construction, breakdown, and repair accompany the bleeding of decidualized endometrium in pseudopregnant mice

Next, we assessed whether morphological changes in overtly bleeding mice are comparable to those associated with human menstruation. We examined the morphology of uterine cross-sections before decidualization, on day 4 of pseudopregnancy, and at various points in time after induction of decidualization (from day 6 to 12).

**Figure 3:**
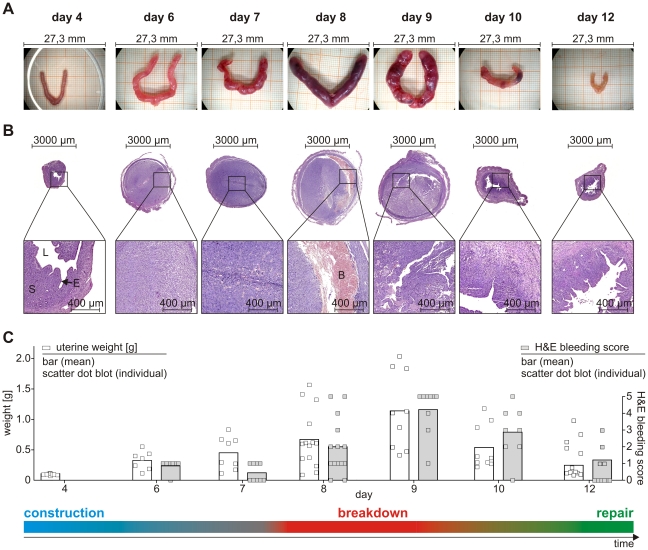
Bleeding pattern and uterine weight mirror changes such as tissue construction, breakdown, and repair. Pseudopregnant BALB/c mice were treated according to Figure 2A and sacrificed before (day 4) or after decidualization (days 6, 7, 8, 9, 10, 12). **A:** Macroscopic analysis of uteri at indicated points in time. **B:** Microscopic analysis of H&E-stained uteri (S, stromal cells; E, epithelium; L, lumen; B, extravasated blood). **C:** Analysis of uterus weights (left axis; open square = individual data; bar = mean) and bleeding intensity determined by H&E scores according to Figure 1 (right axis; filled square and bar analogous to the preceding; N≥7). The two-tailed Spearman test indicates a significant correlation (p = 0.0167, Spearman r = 0.9429) between increased uterus weight and H&E score. Data were assigned to the respective time (day 4 to day 12) and to the dominant processes at that time (construction - blue, breakdown - red, repair - green).

**Figure 4:**
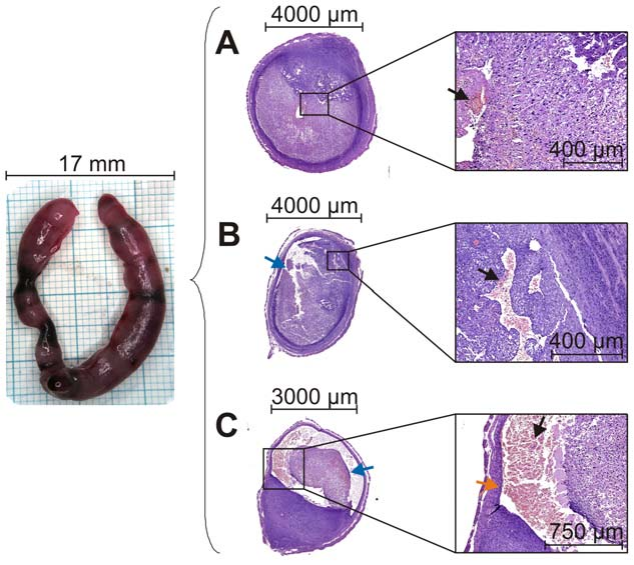
Tissue breakdown and repair are dynamic processes which take place in parallel. Representative H&E-stained sections (right) from the same decidualized uterine horn (left) on day 9 of pseudopregnancy displaying: **A:** Decidualization and first signs of extravasation of blood (black arrow). **B:** Clear signs of destruction in the form of detachment of tissue structures (blue arrow) and extravasation of blood. **C:** Pronounced tissue destruction with marked extravasation of blood combined with signs of re-epithelialization (orange arrow; repair).

Uterus size increased after induction of decidualization, with greatly enlarged uteri at day 8 and day 9, followed by a subsequent decrease (Figure 3A). The uterus weight reflected the macroscopically observed changes (Figure 3C; open squares). Moreover, within this time course the color of the uteri changed from pink to dark red and back to pink (Figure 3A). Microscopic analyses clearly demonstrated the construction, breakdown and repair of the endometrium (Figure 3B). On day 4, i.e. before decidualization, the lumen was open and the epithelium intact. Two to 3 days after the injection of sesame oil (day 6/7) the uteri displayed the typical features of decidualization: closely packed decidualized stromal cells, dense vascularization and closure of the uterine lumen. Clear evidence of tissue breakdown and extravasation of blood was seen by day 8 and day 9. These processes were largely completed by day 10 to 12, when re-epithelialization of the luminal endometrial surface was evident. Over the entire time course, we observed a strong correlation between the bleeding intensity determined by H&E staining of vaginal lavage fluid (Figure 3C; gray, filled squares) and the macro- and microscopically determined breakdown and repair. The Spearman rank correlation coefficient between bleeding intensity (H&E score) and uterine weight indicated a positive correlation (Spearman r = 0.9429) significantly different from “no correlation” (p-value = 0.0167). Visible bleeding was detectable on day 8 and peaked at day 9, the days on which the uteri were largest (Figure 3A), weights were highest (Figure 3C) and tissue breakdown was pronounced (Figure 3B). It was notable that consecutive sections of the same uterine horn displayed different degrees of breakdown and repair (Figure 4). In the uterine section depicted in Figure 4A only marginal extravasation of blood and hardly any obvious signs of degradation or repair were detectable, while the section in Figure 4C showed marked tissue detachment and the first signs of repair, in particular re-epithelialization of the luminal surface. This clearly emphasizes the dynamic nature of the processes.

Overall, decidualized endometrium of pseudopregnant mice displays immense morphological changes: tissue construction, breakdown, extravasation of blood, and repair, accompanied by gain and loss of uterine weight and size, and, most importantly, by overt vaginal bleeding.

### mRNA expression is differentially regulated during cyclic remodeling of the endometrium

**Figure 5:**
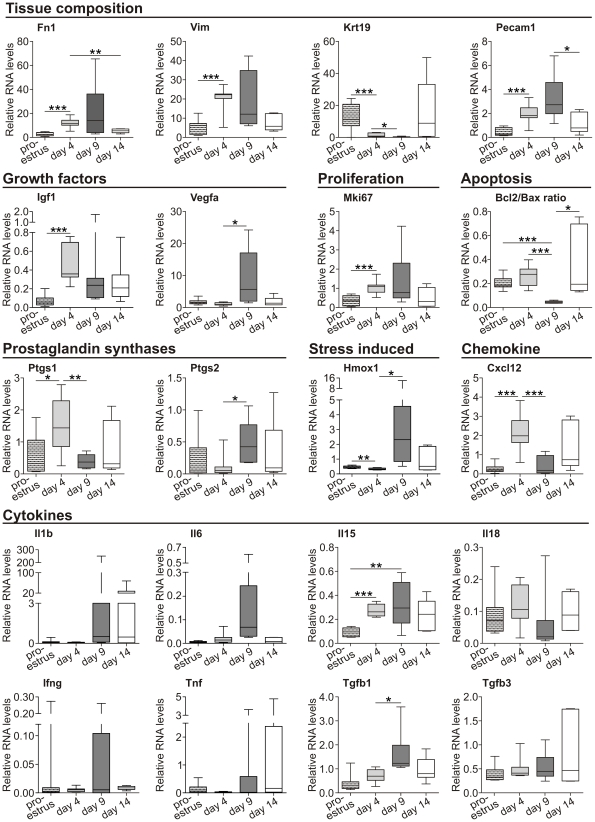
Regulation of different cellular processes in the endometrium is time point-specific. Endometrial mRNA levels of the indicated genes were determined by quantitative real-time PCR. All values were normalized to HPRT mRNA levels. Boxplots summarize the results of 5 to 9 mice per group (unpaired two-tailed Student's t-test, *p≤0.05, **p≤0.01, ***p≤0.001).

Coordination of the various menstrual events is achieved by regulation of a number of genes involved in processes such as decidualization, angiogenesis, proliferation, apoptosis, and inflammation [15]. In order to discover how the menstruation-like bleeding seen in mice correlates with the endometrial expression of genes known to be regulated in human menstruation, we analyzed mRNA levels at different points in time as shown in Figure 5. Endometrial samples were collected from mice in proestrus, on day 4 of pseudopregnancy, and after decidualization on day 9 of pseudopregnancy (i.e. the day on which bleeding was heaviest, see Figure 2, D and E) and on day 14 (i.e. after complete repair).

In general, we identified three different mRNA expression profiles. The first was characterized by a peak in endometrial mRNA expression on day 4 of pseudopregnancy. This was shown for the stromal cell-derived vimentin (VIM), fibronectin 1 (Fn1; a factor of the extracellular matrix), insulin growth factor 1 (Igf1), the proliferation marker Mki67, and the prostaglandin-endoperoxide synthase 1 (Ptgs1; cyclooxygenase-1). The second expression pattern had a typical maximum of expression on day 9; the day of heaviest visible bleeding. By this day significantly increased expression was detected for Pecam1 (platelet endothelial cell adhesion molecule, CD31), which accounts for a large proportion of intercellular junctions of endothelial cells, and for the growth factor Vegfa (vascular endothelial growth factor A). In addition, prostaglandin-endoperoxide synthase 2 (Ptgs2) was up-regulated 10-fold during bleeding (day 9) compared to day 4. It was noteworthy that the expression of heme oxygenase (decycling) 1 (Hmox1), a hypoxia- and inflammatory stress-induced essential enzyme in heme catabolism, was clearly up-regulated during bleeding. A similar expression pattern was also seen for the pro-inflammatory cytokines Il6, Il15, and for the immunosuppressive Tgfb1. In contrast, keratin 19 (Krt19) and Krt8 mRNA (data not shown), both characteristic of epithelial cells, were regulated in the opposite direction and were significantly down-regulated at the time of bleeding (day 9). This third type of expression pattern was also observed for the chemokine Cxcl12 and tendentially for Il18. Interestingly, the ratio of expression of the pro-survival factor Bcl2 to the pro-apoptotic factor Bax was 5.7 times lower during bleeding compared to day 4 of pseudopregnancy. Despite this, we also examined the expression of Il1b, Ifng, Tnf and Tgfb3, which were not differentially regulated.

To sum up, the results imply that genes important for various cell- and tissue-specific processes were differentially regulated within the decidualized endometrium of pseudopregnant mice. In general, three expression patterns were represented. The pattern most frequently detected here was the up-regulation of genes during bleeding on day 9.

## Discussion

Experimentally induced overt menstruation has not been reported for any species so far. Mouse models mimicking menstruation are known, but overt bleeding has never been described [7], [8], [9], [10], [11]. The data presented in this study show that menstruation is not just species-specific. It is more likely that previous experimental approaches have not fulfilled the prerequisites for overt menstruation. Our data confirmed that pseudopregnant mice with decidualized endometrium display endometrial tissue destruction and repair. Moreover, for the first time, active flushing of shed tissue and blood from the vagina as a feature of overt menstruation was observed in mice. Uterine bleeding was triggered by the natural drop of previously increased endogenous progesterone, which occurred between day 7 and day 8 or day 8 and day 9 of pseudopregnancy. This temporal variation may reflect the individuality of mice with respect to the degradation of their corpora lutea in the ovary. Withdrawal of endogenous progesterone coincided with the onset of bleeding. The causal relationship of both events was confirmed by experimental antagonizing endogenous progesterone signaling through mifepristone administration that apparently caused almost immediate bleeding (12 h later).

The induction of overt bleeding in a naturally non-menstruating species like the mouse support the hypothesis that the amount of tissue and blood determines whether expulsion or reabsorption takes place [13]. It was possible to achieve differentiation of a large amount of tissue by increasing the stimulus for decidualization in the form of a 5-fold larger volume of oil than that used by other authors [8], [9], [10], [11], [12]. This led to decidualization of both uterine horns, which further increased the amount of tissue and blood to be flushed out. In order to obtain overt bleeding, we profited from the intrinsic ovarian regulation of intact pseudopregnant female mice compared to ovariectomized mice supplied by exogenous hormones used in former studies [7], [8], [9], [11], [12]. In comparison to the established model, we prolonged the duration of the processes underlying bleeding: changes in uterus size and morphology occurred very rapidly within hours after progesterone withdrawal in ovariectomized mice, whereas these processes took place over a few days in our study [8], [12]. This extension of time, together with the determination of heavy bleeding by scoring bleeding intensity, will increase the feasibility of analyzing the kinetics of bleeding or its intensity. Hence, overtly bleeding mice are expected to be valuable for studying the molecular pathways affecting menstrual disorders such as menorrhagia.

**Figure 6:**
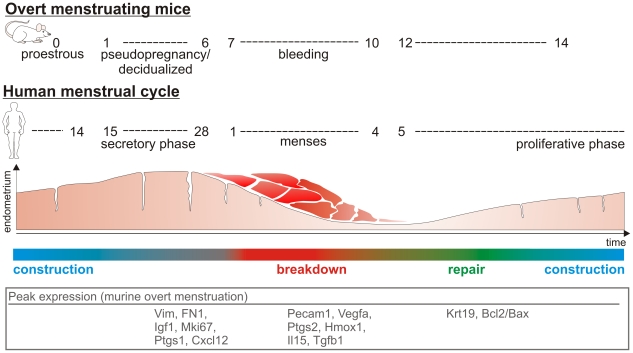
Overt bleeding in mice mimics the human menstrual cycle. Comparison of the overtly bleeding mice presented here and the human menstrual cycle. The proestrus, with high levels of estrogen, might mimic the proliferative phase in humans. Induction of pseudopregnancy causes progesterone levels to increase in the same way as in the human secretory phase. The drop of progesterone induces bleeding between day 7 and day 10, which is comparable to menses. By day 12 tissue repair is well advanced, something which is also seen in the early proliferative phase. These processes are governed by differential expression of various genes. Genes that were expressed at a maximum at certain time points in overtly bleeding mice are listed. The vast majority of them are regulated similarly in mice and humans (for details see text).

Our data demonstrate that the conversion of non-menstruating mice to mice displaying overt bleeding as a characteristic of menstruation is feasible. This observation argues against the hypothesis put forward by Profet that menstruation is a mechanism for pathogen defense [2]. The induction of menstruation in a species that does not normally menstruate and in the absence of any modification of the number of sexual contacts refutes the assumption that the copiousness of menstruation increases with the promiscuity of the mating system. Rather, our data indicate that it is a matter of costs and benefits: where there are large quantities of decidualized endometrial tissue, as was found in the mice in this study, it seems to be metabolically more economical to repel tissue rather than to absorb it. Taking both into account - the morphological uterine changes demonstrated here and the variations in hormone levels (i.e. progesterone) - we propose a functional correlation between overtly menstruating mice and human menstruation as shown in Figure 6. The proestrus in mice might represent the proliferative phase in women, since estrogen is the dominant hormone at this time. After induction of pseudopregnancy, however, progesterone levels are greatly increased, indicating the similarities to the human secretory phase. The bleeding between day 7 and day 10 which is triggered by the drop in progesterone mimics menses, and day 12 corresponds to tissue repair in the early proliferative phase. Indeed, similar regulation of marker genes also supports the proposed link between overtly bleeding mice and human menstruation. Fn1 mRNA was increased in the “secretory-like” phase on day 4 (to day 9) of pseudopregnancy in line with human progestin-stimulated FN1 expression [16]. Increased expression of stromal cell-derived vimentin during the human secretory phase and on day 4 (to day 9) of mouse pseudopregnancy might account for the cyclic variations in tissue composition in both species [17]. The significant down-regulation (bleeding on day 9) and subsequent up-regulation (day 14) of the epithelium-derived keratin 19 mRNA (and Krt8, data not shown) is thought to be a molecular sign of ongoing epithelial breakdown and repair as suggested by immunohistological investigations [10]. In addition, the expression of Pecam1 (CD31) was increased during bleeding in mice, and PECAM1 is intensely stained in stroma and endothelial cells during human menstruation [18]. Correspondingly, the mRNA of the pro-angiogenic factor VEGF-A, which is highly up-regulated in human menstruation, reached its highest level of expression during bleeding in mice [19], [20]. The growth activity of stroma cells is elevated in both the proliferative phase and the late secretory phase of the menstrual cycle, whereby the latter is induced by progesterone in a second wave of proliferation [21], [22]. By investigating the expression of Mki-67 mRNA which is expressed by cycling cells, we demonstrated progesterone-induced proliferation on day 4 of pseudopregnancy in mice [23]. Moreover, humans display cyclic mRNA expression of the pro-survival factor BCL-2 [24]. A contribution of apoptosis was likewise demonstrated in menstruation-like processes in mice. The Bcl2/Bax ratio was exclusively decreased during bleeding as an indicator of the regulation of survival and apoptosis. Whether Hmox1 displays cycle-dependent expression during human menstruation is not completely understood [25], [26]. However, Hmox1 mRNA is typically induced after erythrocyte lysis and in hypoxia and inflammation, suggesting increased expression during tissue breakdown and bleeding which was confirmed by our data. Menstrual breakdown is triggered by prostaglandins, and both rate-limiting enzymes of prostaglandin synthesis, PTGS1 and PTGS2, display cyclic expression [27]. The high expression of PTGS1 on day 4 of pseudopregnancy (human secretory phase) and increased expression of PTGS2 during bleeding (human menstruation) are consistent with human data [27].

It is widely accepted that immunological mediators play a role in menstruation and, correspondingly, we detected regulated gene expression for pro-inflammatory cytokines like Il6 and Il15, as has been described in humans [28], [29], [30]. As in another mouse study and in human endometrium, the data point tendentially towards cyclic Il18 expression [31], [32]. A similar scenario with ambiguous results from human endometrial biopsies was found for CXCL12 (SDF-1) [33], [34], [35]. Interestingly, Laird *et al.* describe a CXCL12 mRNA expression pattern (with a tendency towards greater expression in the early and late secretory phase) in human endometrium that appears to be comparable to the one seen in the overtly bleeding mice [35]. Concerning the Tgfb cytokine family, which is important for the induction of wound healing, we observed partial correlation of our data with human data: TGFB1 mRNA is increased during the menstrual phase in humans, corresponding to the expression pattern observed in this study [36], [37], [38]. In contrast, average TGFB3 mRNA levels were reported to be increased 3-fold from the human secretory to the menstrual phase, but were fairly constant in our study [37].

In principle, when translating gene expression, histology, and bleeding data from mice to women or vice versa one must consider that human endometrial biopsies are taken over a period of days (e.g. for menstruation between day 28 and day 5 of the cycle) and not at a clearly defined point in time as was the case in this study [37]. Our data demonstrate that the decidualized endometrium is a highly dynamic tissue in which processes such as tissue destruction and repair proceed in parallel. Accordingly, it must be expected that changes occur within a very short time frame in humans as well, especially during menstruation. The exact time at which tissue is collected will therefore determine the results obtained and is decisive for the transferability of data from mouse to man.

In conclusion, the comparison of overtly bleeding mice and human data for menstruation indicates a very strong correlation, not only in terms of morphology and hormones but also regarding the common regulation of marker genes, thus indicating comparable underlying processes. Since intact pseudopregnant mice convincingly mimic human menstruation, it is expected that the further use of these mice will contribute to a better understanding of the cellular and molecular mechanisms accompanying menstruation. Moreover, they will be a valuable tool for pharmacologic testing.
